# Draft genome sequence of *Exiguobacterium* sp. from whole cantaloupe, with inhibition capacity against *Listeria monocytogenes*

**DOI:** 10.1128/mra.00850-23

**Published:** 2023-12-14

**Authors:** Tatiana A. Vishnivetskaya, Jeffrey Niedermeyer, Eduardo Guttierrez-Rodriguerz, David Baltzegar, Cameron Parsons, Sophia Kathariou

**Affiliations:** 1 Department of Microbiology, University of Tennessee, Knoxville, Tennessee, USA; 2 Department of Food Science, North Carolina State University, Raleigh, North Carolina, USA; 3 Department of Horticulture and Landscape Architecture, Colorado State University, Fort Collins, Colorado, USA; 4 Genomic Sciences Laboratory, North Carolina State University, Raleigh, North Carolina, USA; University of Southern California, Los Angeles, California, USA

**Keywords:** *Exiguobacterium*, genome, inhibition of Listeria monocytogenes

## Abstract

We report the draft genome sequence of a novel species, *Exiguobacterium* sp., isolated from a freshly harvested and untreated cantaloupe in North Carolina. The strain *Exiguobacterium* wild type exhibited inhibitory activity against the foodborne pathogen *Listeria monocytogenes*, including strains of diverse serotypes and genotypes, both on agar media and in biofilms.

## ANNOUNCEMENT

**Fig 1:**
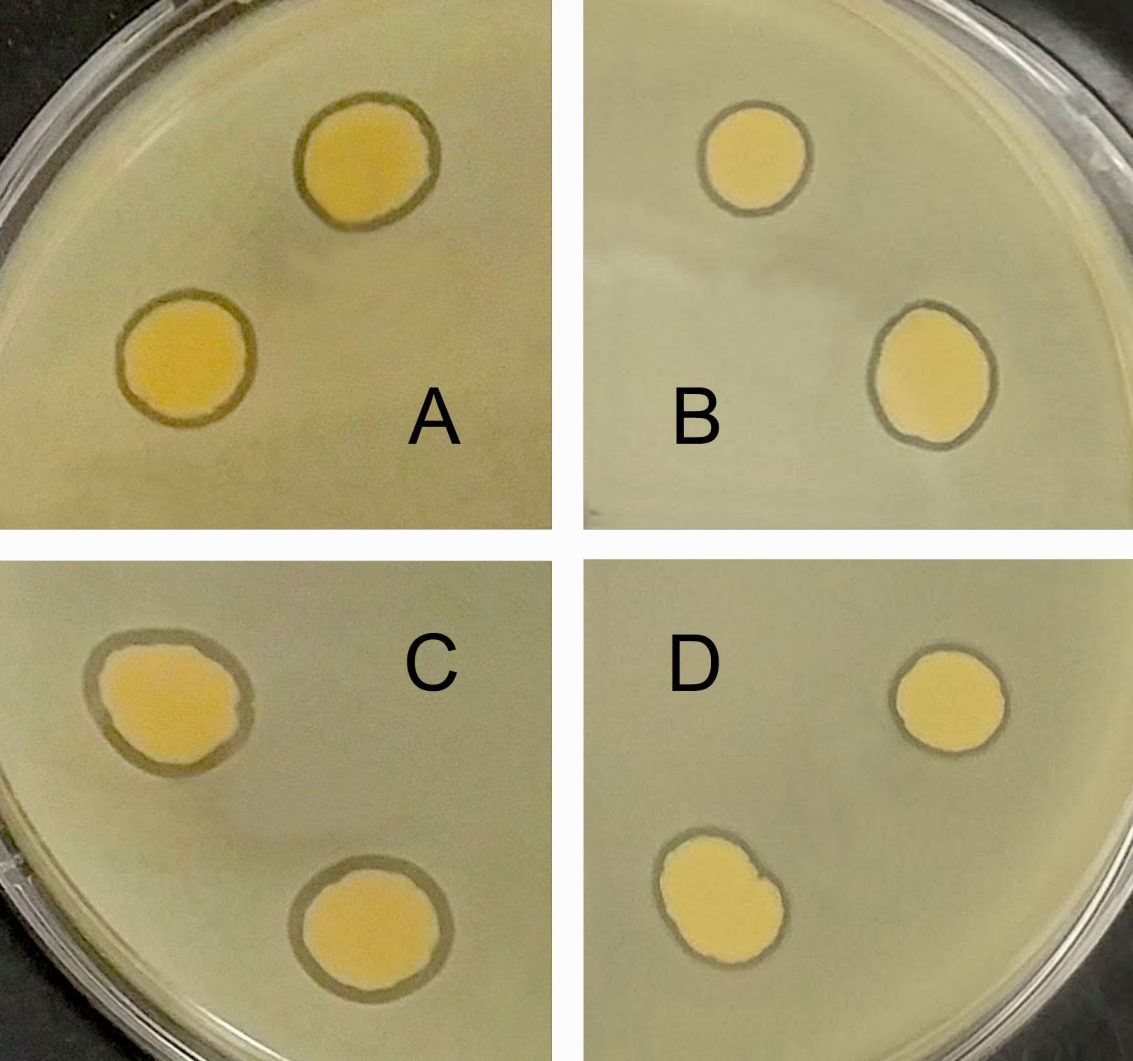
Inhibition zones produced by *Exiguobacterium* WT on lawns of *L. monocytogenes*. The inhibition zones are visible as dark rings around the spots of *Exiguobacterium* WT on the lawn of *L. monocytogenes*. The *L. monocytogenes* lawn is pale yellow, while the *Exiguobacterium* WT spots are darker yellow. Assays were done as described (6). Briefly, *Exiguobacterium* WT was spotted (5 µL) on soft (0.4%) TS agar mixed with *L. monocytogenes* 2011L-2858 (serotype 1/2b, panels A and B) and incubated at (**A**) 25°C, 48 h, and (**B**) 37°C, 24 h, or with *L. monocytogenes* F8027 (serotype 4b, panels C and D) and incubated at (**C**) 25°C, 48 h, and (**D**) 37°C, 24 h. *L. monocytogenes* and *Exiguobacterium* WT used for the lawns and the spots, respectively, were grown in TS broth for 24 h at the temperatures employed for the incubations.

*Exiguobacterium* is a large, versatile, and ubiquitous genus (1, 2). Frequent association with fresh produce (3) makes *Exiguobacterium* spp. promising candidates as biocontrol agents for inhibition of the bacterial foodborne pathogen *Listeria monocytogenes*, implicated in produce-related outbreaks (4). A major outbreak of listeriosis in the USA in 2011 involved whole cantaloupe (4, 5). Whole, ripe cantaloupe (*Cucumis melo*) was harvested from a field in North Carolina, USA (35°10′28″N, 77°48′44″W) and transported to North Carolina State University. Upon arrival at the laboratory, phosphate-buffered saline rinsates of the fruit were prepared and analyzed as described (6) for inhibition of *L. monocytogenes* 2011L-2858, derived from the 2011 cantaloupe outbreak (4), on soft (0.4%) trypticase soy (TS) agar (BD, Sparks, MD, USA). Zones of inhibition (~1.5 mm) were detected around light-yellow colonies of Gram-positive, rod-shaped bacteria, which were subsequently purified on TS agar. Analysis of 16S rRNA sequences using colony PCR and universal primers 8F and 1492R (7) indicated that the isolate is most closely related to *Exiguobacterium acetylicum* AMCC 101217 (CP030931.1) at 92% identity, and the strain was designated *Exiguobacterium* wild type (WT). Further tests done as described (6) showed that *Exiguobacterium* sp. WT inhibits growth (Fig. 1) of all tested strains of *L. monocytogenes* serotypes 1/2a, 1/2b and 4b, responsible for most human listeriosis (8).

Genomic DNA (gDNA) was extracted from *Exiguobacterium* WT grown in TS broth at 37°C overnight with the DNeasy Blood and Tissue kit (Qiagen, Valencia, CA, USA). The gDNA was quantified by a Qubit v.2.0 fluorometer and an Agilent 2200 TapeStation high molecular weight DNA assay. Paired-end libraries were prepared using 200 ng of gDNA with a TruSeq Nano DNA Library Prep (Illumina, San Diego, CA, USA). Briefly, the gDNA fragments, obtained using a Covaris S220 Ultrasonicator and purified using AMPure XP beads, were end-repaired followed by 550-bp insert size selection and enriched by PCR amplification. Quality and concentration of the amplified library was checked using an Agilent 2200 TapeStation (D1000) before sequencing on an Illumina NovaSeq 6000, utilizing a shared XP lane of 2 × 150-bp paired-end S4 flow cell reagent kit (Illumina). Data quality was checked using FastQC v.0.11.9 (9). Default parameters were used for all software unless otherwise specified. Genome assembly utilized SPAdes v.3.13.1 (10). Annotation of genome assembly and putative pathways was done by the NCBI PGAP v.6.6 (11) (Table 1).

**TABLE 1:** Assembly and annotation metrics

Feature	*Exiguobacterium* WT from cantaloupe
No. of quality-filtered paired-end reads	16,520,591
No. of contigs	16
*N*_50_ (bp)	2,106,256
Largest scaffold length (bp)	2,106,256
Assembly length (bp)	3,105,902
Average genome coverage (×)	797
G + C content (%)	46.63
No. of genes	3,215
No. of coding DNA sequences	3,163
No. of 16S rRNA genes	7
No. of RNA genes	52
No. of tRNA genes	42
No. of pseudogenes	16
Transposase	1
Biosynthetic gene clusters	3
Phages and prophages	8

Whole-genome sequence analysis indicated that the *Exiguobacterium* WT had the closest similarity to the plant-associated strains Leaf196 and RIT341 at 98.26% average nucleotide identity (ANI), and 91.12% ANI to the type strain *E. acetylicum* DSM20416. Considering the strain’s inhibitory potential against *L. monocytogenes*, we subjected the genome to *in silico* secondary metabolite analysis using antiSMASH v.5.0 (12) and antibiotic resistance target seeker (ARTS v.2.0) (13). The genome harbored a putative biosynthetic pathway for the production of a toyoncin-like peptide active against the foodborne pathogens *Bacillus cereus* and *L. monocytogenes* (14). The genome contains two genes encoding L-alanyl-D-glutamate peptidase, an endolytic enzyme that degrades peptidoglycan and causes hydrolysis of the cell wall in *L. monocytogenes* and other bacteria (15). *Exiguobacterium* WT from cantaloupe fruit may represent a promising candidate for development of novel strategies to control *L. monocytogenes* in the food supply.

## Data Availability

This Whole Genome Shotgun project has been deposited at DDBJ/ENA/GenBank under accession number JAVFVO000000000. The version described in this paper is version JAVFVO010000000. The raw sequencing reads have been submitted to Sequence Read Archive under accession number SRP465708.

## References

[1] Vishnivetskaya TA , Kathariou S , Tiedje JM . 2009. The exiguobacterium genus: biodiversity and biogeography. Extremophiles 13:541–555. doi:10.1007/s00792-009-0243-5 19381755

[2] Kasana RC , Pandey CB . 2018. Exiguobacterium: an overview of a versatile genus with potential in industry and agriculture. Crit Rev Biotechnol 38:141–156. doi:10.1080/07388551.2017.1312273 28395514

[3] Hu A , Gao C , Lu Z , Lu F , Kong L , Bie X . 2021. Detection of Exiguobacterium spp. and E. acetylicum on fresh-cut leafy vegetables by a multiplex PCR assay. J Microbiol Methods 180:106100. doi:10.1016/j.mimet.2020.106100 33249127

[4] Garner D , Kathariou S . 2016. Fresh produce-associated listeriosis outbreaks, sources of concern, teachable moments, and insights. J Food Prot 79:337–344. doi:10.4315/0362-028X.JFP-15-387 26818997

[5] McCollum JT , Cronquist AB , Silk BJ , Jackson KA , O’Connor KA , Cosgrove S , Gossack JP , Parachini SS . 2013. Multistate outbreak of listeriosis associated with cantaloupe. N Engl J Med 369:944–953. doi:10.1056/NEJMoa1215837 24004121

[6] Reina LD , Breidt J , Fleming HP , Kathariou S . 2005. Isolation and selection of lactic acid bacteria as biocontrol agents for nonacidified, refrigerated pickles. J Food Sci:M7–M11. doi:10.1111/j.1365-2621.2005.tb09050.x

[7] Baker GC , Smith JJ , Cowan DA . 2003. Review and re-analysis of domain-specific 16S primers. J Microbiol Methods 55:541–555. doi:10.1016/j.mimet.2003.08.009 14607398

[8] Swaminathan B , Gerner-Smidt P . 2007. The epidemiology of human listeriosis. Microbes Infect 9:1236–1243. doi:10.1016/j.micinf.2007.05.011 17720602

[9] Andrews S . 2010. FastQC: a quality control analysis tool for high throughput sequencing data. Available from: https://githubcom/s-andrews/FastQC

[10] Bankevich A , Nurk S , Antipov D , Gurevich AA , Dvorkin M , Kulikov AS , Lesin VM , Nikolenko SI , Pham S , Prjibelski AD , Pyshkin AV , Sirotkin AV , Vyahhi N , Tesler G , Alekseyev MA , Pevzner PA . 2012. Spades: a new genome assembly algorithm and its applications to single-cell sequencing. J Comput Biol 19:455–477. doi:10.1089/cmb.2012.0021 22506599 PMC3342519

[11] Li W , O’Neill KR , Haft DH , DiCuccio M , Chetvernin V , Badretdin A , Coulouris G , Chitsaz F , Derbyshire MK , Durkin AS , Gonzales NR , Gwadz M , Lanczycki CJ , Song JS , Thanki N , Wang J , Yamashita RA , Yang M , Zheng C , Marchler-Bauer A , Thibaud-Nissen F . 2021. Refseq: expanding the prokaryotic genome annotation pipeline reach with protein family model curation. Nucleic Acids Res 49:D1020–D1028. doi:10.1093/nar/gkaa1105 33270901 PMC7779008

[12] Blin K , Shaw S , Steinke K , Villebro R , Ziemert N , Lee SY , Medema MH , Weber T . 2019. antiSMASH 5.0: updates to the secondary metabolite genome mining pipeline. Nucleic Acids Res 47:W81–W87. doi:10.1093/nar/gkz310 31032519 PMC6602434

[13] Alanjary M , Kronmiller B , Adamek M , Blin K , Weber T , Huson D , Philmus B , Ziemert N . 2017. The antibiotic resistant target seeker (ARTS), an exploration engine for antibiotic cluster prioritization and novel drug target discovery. Nucleic Acids Res 45:W42–W48. doi:10.1093/nar/gkx360 28472505 PMC5570205

[14] Wang J , Xu H , Liu S , Song B , Liu H , Li F , Deng S , Wang G , Zeng H , Zeng X , Xu D , Zhang B , Xin B , Elkins CA . 2021. Toyoncin, a novel leaderless bacteriocin that is produced by Bacillus toyonensis XIN-Yc13 and specifically targets B. cereus and Listeria monocytogenes. Appl Environ Microbiol 87:e0018521. doi:10.1128/AEM.00185-21 33811023 PMC8174769

[15] Korndörfer IP , Kanitz A , Danzer J , Zimmer M , Loessner MJ , Skerra A . 2008. Structural analysis of the L-alanoyl-D-glutamate endopeptidase domain of Listeria bacteriophage endolysin Ply500 reveals a new member of the LAS peptidase family. Acta Crystallogr D Biol Crystallogr 64:644–650. doi:10.1107/S0907444908007890 18560152

